# Curious Epitaph

**Published:** 1854-03

**Authors:** 


CURIOUS EPITAPH.—In a country grave-yard in New Jersey there is a plain stone erected over the grave of a beautiful young lady, with only this inscription upon it:

"Julia Adams, died of thin shoes, April 17, 1839, aged 19."

